# Gender-linked impact of epicardial adipose tissue volume in patients who underwent coronary artery bypass graft surgery or non-coronary valve surgery

**DOI:** 10.1371/journal.pone.0177170

**Published:** 2017-06-08

**Authors:** Gulinu Maimaituxun, Michio Shimabukuro, Hotimah Masdan Salim, Minoru Tabata, Daisuke Yuji, Yoshihisa Morimoto, Takeshi Akasaka, Tomomi Matsuura, Shusuke Yagi, Daiju Fukuda, Hirotsugu Yamada, Takeshi Soeki, Takaki Sugimoto, Masashi Tanaka, Shuichiro Takanashi, Masataka Sata

**Affiliations:** 1 Department of Cardiovascular Medicine, Institute of Biomedical Sciences, Tokushima University Graduate School, Tokushima, Japan; 2 Department of Cardio-Diabetes Medicine, Institute of Biomedical Sciences, Tokushima University Graduate School, Tokushima, Japan; 3 Department of Diabetes, Endocrinology and Metabolism, School of Medicine, Fukushima Medical University, Fukushima, Japan; 4 Department of Cardiovascular Surgery, Sakakibara Heart Institute, Tokyo, Japan; 5 Department of Cardiovascular Surgery, Shonan-Kamakura General Hospital, Kamakura, Japan; 6 Division of Cardiovascular Surgery, Awaji Medical Center, Hyogo, Japan; 7 Cardiology and Catheterization Laboratories, Shonan-Kamakura General Hospital, Kamakura, Japan; The University of Hong Kong, HONG KONG

## Abstract

**Background:**

Traditional and non-traditional risk factors for atherosclerotic cardiovascular disease (ASCVD) are different between men and women. Gender-linked impact of epicardial adipose tissue volume (EATV) in patients undergoing coronary artery bypass grafting (CABG) remains unknown.

**Methods:**

Gender-linked impact of EATV, abdominal fat distribution and other traditional ASCVD risk factors were compared in 172 patients (men: 115; women: 57) who underwent CABG or non-coronary valvular surgery (non-CABG).

**Results:**

In men, EATV, EATV index (EATV/body surface area) and the markers of adiposity such as body mass index, waist circumference and visceral fat area were higher in the CABG group than in the non-CABG group. Traditional ASCVD risk factors were also prevalent in the CABG group. In women, EATV and EATV index were higher in the CABG group, but other adiposity markers were comparable between CABG and non-CABG groups. Multivariate logistic regression analysis showed that in men, CABG was determined by EATV Index and other ASCVD risk factors including hypertension, dyslipidemia, adiponectin, high sensitive C-reactive protein (hsCRP) and type 2 diabetes mellitus (Corrected R^2^ = 0.262, p < 0.0001), while in women, type 2 diabetes mellitus is a single strong predictor for CABG, excluding EATV Index (Corrected R^2^ = 0.266, p = 0.005).

**Conclusions:**

Our study found that multiple risk factors, including epicardial adipose tissue volume and traditional ASCVD factors are determinants for CABG in men, but type 2 diabetes mellitus was the sole determinant in women. Gender-specific disparities in risk factors of CABG prompt us to evaluate new diagnostic and treatment strategies and to seek underlying mechanisms.

## Introduction

Traditional and non-traditional risk factors for atherosclerotic cardiovascular disease (ASCVD) are different between men and women, and such differences may alter the clinical course of ASCVD [1–5]. Among traditional ASCVD risk factors, obesity and overweight are becoming more prevalent in the world, and considerably affect the onset of ASCVD [6]. Current evidence indicate that distribution of abdominal fat and non-abdominal fat is strongly associated with ASCVD [7]. Although the distribution of abdominal and non-abdominal fat differs largely between men and women [8, 9], the gender-specific difference has not been fully evaluated in ASCVD.

Recently, epicardial adipose tissue (EAT), which belongs to non-abdominal fat, has gathered scientific interest, as it may be a marker or a pathophysiologic source of ASCVD [10–12]. In 2,751 participants without known coronary artery disease (CAD), EAT volume showed an odds ratio of 1.74 (95% CI 1.0 to 2.95 for each doubling of EAT) for major adverse cardiac events (MACE), even after adjustment for the Framingham Risk Score, coronary calcium score and body mass index [13]. It is known that EAT volume is more significantly and directly correlated with accumulation of visceral adipose tissue (VAT) compared to subcutaneous adipose tissue (SAT) volume [11]. Since VAT is deposited preferably in men than in women, one can hypothesize that accumulation of EAT affects ASCVD largely in men. However, it remains unclear whether increased EAT volume indicated an independent and additive risk when VAT mass is accounted for and whether such notion differs between men and women.

We compared the gender-linked impact of EAT volume, taking into account abdominal fat distribution and traditional ASCVD risk factors, in patients undergoing coronary artery bypass grafting (CABG) with that in patients undergoing non-coronary valvular surgery.

## Methods

### Study populations

This study had been originally designed to collect samples of epicardial and subcutaneous fat pad and compare its profiles between patients with coronary artery and non-coronary artery diseases [10]. We thereafter increased sampling institutions and recruited 172 patients (men: 115; women: 57), who underwent coronary artery bypass graft surgery (CABG group) or non-coronary valvular surgery (non-CABG group) from July 2009 to September 2014 at: Department of Cardiovascular Surgery, Sakakibara Heart Institute, Tokyo, Japan; Department of Cardiovascular Surgery, Shonan-Kamakura General Hospital, Kamakura, Japan; and Division of Cardiovascular Surgery, Awaji Medical Center, Hyogo, Japan. Patients underwent 256-slice multi-detector computed tomography (MDCT) before CABG or valvular surgery. The protocol of this study was approved by the institutional review boards of the University of Tokushima Hospital, Sakakibara Heart Institute; Shonan-Kamakura General Hospital, and Awaji Medical Center. All subjects gave their written informed consent before beginning the study. All participants provided written informed consent after they were advised regarding the radiation exposure-related risk and possible complications of iodine-containing contrast. Exclusion criteria included patients with iodine-based contrast allergy or renal failure (creatinine > 1.5 mg/ml). Hypertension was defined as a systolic blood pressure of ≥ 140 mmHg and/or diastolic blood pressure of ≥ 90 mmHg, or as the current use of antihypertensive medication. Diabetes was defined as HbA1c concentration ≥ 6.5% or fasting plasma glucose level > 126 mg/dL, or the current use of antidiabetic medication. Dyslipidemia was defined as either of serum low-density lipoprotein (LDL) cholesterol level of ≥ 140 mg/dL, serum triglyceride (TG) level ≥ 150 mg/dL and serum high-density lipoprotein (HDL) cholesterol level < 40 mg/dL and/or the current use of anti-hyperlipidemic medication. Metabolic syndrome was defined as presence of abdominal obesity (modified Japanese Criteria; waist circumference: men ≥ 90 cm; women ≥ 80 cm), plus any two of the following four factors: 1. hypertriglyceridemia (serum triglyceride (TG) level ≥ 150 mg/dL (1.69 mmol/L); 2. low high-density lipoprotein (HDL) cholesterol (serum level < 40 mg/dL (1.04 mmol/L); 3. elevated blood pressure: systolic blood pressure ≥ 130 mmHg or diastolic blood pressure ≥ 85 mmHg; 4. high fasting glucose (serum level ≥ 100 mg/dL). All subjects were categorized into the CABG group or non-CABG group (non-coronary valvular surgery). Glycated hemoglobin A1C (HbA1c) concentration was measured by high-performance liquid chromatography and insulin level by chemiluminescent enzyme immunoassay. The value of HbA1c [Japan Diabetic Society (JDS) %] was converted to National Glycohemoglobin Standardization Program (NGSP) levels using the formula HbA1c (NGSP %) = HbA1c (JDS %) + 0.4%, considering the relational expression of HbA1c (JDS %) concentration measured by the Japanese standard [14]. Serum concentrations of high sensitive C-reactive protein (hsCRP) and adiponectin were measured as described [15].

### Multi-detector CT scan protocol and analysis of EAT

Multi-detector CT scan was performed as previously described [10]. Briefly, for the measurement of EATV, volume measurement software of Vincent was employed to detect EATV. Volumetric measurements were performed on axial views of 5 mm slice thickness and number of slices ranging between 300 and 320; non-contrast cardiac computed tomography (CT) is the method of choice for quantification of EAT volume from the surrounding tissue based on density differences. In a semi-automated process, the pericardium counter is first manually traced in each trans-axial slice, followed by an automated step of processing all continuous voxels with a density range of -190 to -30 Hounsfield Units (HU) within the pericardial sac, for calculation of EAT volume. The upper border of the EATV measurements was the lower surface of the left pulmonary artery origin, and the lower border was the left ventricle apex. The area of EAT surrounding the proximal, middle, and distal segments of the major coronary arteries was included in the volumetric measurements. Region of interest was placed within the visceral epicardium and volume of adipose tissue was calculated. A density ranges of -190 to -30 HU was used to isolate the adipose tissue. The EAT area of each slice was then summed and multiplied by the slice thickness and number of slices to determine the total volume of EAT. Anthropometric measurements were made in the standing position. The subcutaneous fat area (SFA) and intra- abdominal visceral fat area (VFA) were measured at the level of the umbilicus, using a standardized method with CT scans as previously described [15].

### Assessment of coronary atherosclerosis

Coronary angiography was performed by a standard technique. To prevent coronary spasm, all patients received intracoronary injection of isosorbide dinitrate at an optimal dose before angiography. Two independent observers blinded to the results of the analyses reviewed the coronary angiograms separately. Stenosis >75% detected angiographically in a major coronary vessel was defined as significant stenosis. Gensini scores were calculated based on angiographic findings, as previously reported [16].

### Statistical analysis

The data for the continuous variables with a normal distribution are expressed as mean ± SD, using the unpaired two-sample T-test for comparison of the means in the two groups. The data for the nominal variables are expressed with Chi-square test. Multiple logistic regression analysis was done to adjust for confounding factors. Variables are treated as continuous: 1 represents a risk and 0 represents no risk. Multivariate logistic regression analysis was used to evaluate the EATV Index determinants, by dividing the groups into men and women, after adjustment for age, body mass index (BMI), CABG, hypertension, dyslipidemia, and type 2 diabetes mellitus. Multivariate logistic regression analysis was adopted to evaluate the association between EATV index and its impact on CABG in men and women, after adjustment for age, BMI, hypertension, dyslipidemia, type 2 diabetes mellitus and EATV Index. Statistical analysis was performed using Stat view or JMP 12.2.0 (SAS Institute Inc. Cary, NC, U.S.A.). P < 0.05 was considered to be statistically significant.

## Results

### General characteristics

General characteristics of the study patients are shown in **Table 1**. In men, although age was comparable, body weight, BMI, waist circumference (WC) and VFA were higher in the CABG group than in the non-CABG group (see *P1*, **Table 1**). Epicardial adipose tissue volume (EATV) and EATV/body surface area (EATV Index) were higher in the CABG group. Diastolic blood pressure, hypertension (%), plasma glucose, HbA1c concentration, type 2 diabetes mellitus (%), LDL cholesterol levels, LDL cholesterol level ≥ 140 mg/dL or statin use, triglyceride level, and TG level ≥ 150 mg/dL or HDL level < 40 mg/dL were higher, and HDL cholesterol level was lower in the CABG group than in the non-CABG group. Prevalence of type 2 diabetes mellitus, hypertension, and dyslipidemia (LDL cholesterol level ≥ 140 mg/dL or statin use, and TG level ≥ 150 mg/dL or HDL-cholesterol level < 40 mg/dL) were higher in the CABG group. hsCRP level was higher and adiponectin level was lower in the CABG group.

**Table 1:** General characteristics of studied patients.

	Men (n = 115)					Women (n = 57)						
	non-CABG (n = 47)		CABG (n = 68)			non-CABG (n = 40)			CABG (n = 17)			
Parameters	mean or %	(SD)	mean or %	(SD)	*P1*	mean or %	(SD)	*P2*	mean or %	(SD)	*P1*	*P2*
Age (years)	67	(12)	66	(11)	0.786	72	(10)	0.044	76	(7)	0.158	0.002
Body weight (kg)	61.6	(9.6)	67.5	(12.2)	0.007	50.0	(8.7)	0.000	47.3	(8.4)	0.308	0.000
Body mass index (kg/m^2^)	23.0	(3.0)	24.6	(3.8)	0.013	22.2	(3.3)	0.245	21.0	(2.9)	0.202	0.000
Body mass index ≥ 25 kg/m^2^ (%)	34%	-	41%	-	0.559	30%	-	0.819	18%	-	0.513	0.094
Waist circumference (cm)	85.2	(9.6)	90.4	(10.9)	0.008	81.3	(10.4)	0.074	81.4	(11.5)	0.971	0.003
Visceral fat area (cm^2^)	125	(64)	155	(75)	0.027	88	(46)	0.003	91	(48)	0.823	0.001
Subcutaneous fat area (cm^2^)	110	(59)	136	(76)	0.052	133	(88)	0.149	122	(67)	0.647	0.497
EATV (cm^3^)	91	(57)	131	(79)	0.003	85	(54)	0.639	132	(66)	0.007	0.978
EATV index (cm^3^/m^2^)	53	(35)	74	(40)	0.004	60	(42)	0.369	93	(43)	0.009	0.085
Systolic blood pressure (mmHg)	126	(20)	129	(19)	0.443	120	(17)	0.191	127	(14)	0.282	0.717
Diastolic blood pressure (mmHg)	64	(14)	71	(13)	0.017	68	(13)	0.274	67	(13)	0.807	0.356
Hypertension (%)	75%	-	93%	-	0.014	75%	-	1.000	100%	-	0.025	0.578
Smoking history (%)	70%	-	71%	-	1.000	23%	-	0.000	24%	-	1.000	0.001
Fasting plasma glucose (mg/dL)	109	(29)	139	(49)	0.001	112	(22)	0.628	127	(36)	0.113	0.460
HBA1C (%)	5.59	(0.73)	6.17	(0.97)	0.001	5.70	(0.44)	0.424	6.64	(1.07)	0.000	0.102
Type 2 diabetes mellitus (%)	26%	-	53%	-	0.004	33%	-	0.488	82%	-	0.001	0.031
LDL cholesterol (mg/dL)	101	(26)	98	(30)	0.623	113	(27)	0.041	111	(27)	0.781	0.148
LDL cholesterol ≥ 140 mg/dL or statin use (%)	32%	-	60%	-	0.004	48%	-	0.186	71%	-	0.149	0.578
HDL cholesterol (mg/dL)	55	(17)	46	(13)	0.002	63	(16)	0.032	48	(13)	0.002	0.611
Triglyceride (mg/dL)	120	(85)	152	(86)	0.055	113	(67)	0.645	174	(130)	0.022	0.407
TG≥150mg/dL or HDL<40mg/dL (%)	25%	-	54%	-	0.003	25%	-	1.000	50%	-	0.112	0.787
hsCRP (mg/dL)	1.04	(2.57)	0.37	(0.86)	0.047	0.72	(1.82)	0.514	1.25	(2.83)	0.400	0.028
Adiponectin (μg/mL)	9.15	(10.5)	5.89	(5.38)	0.033	11.17	(9.17)	0.352	6.96	(3.95)	0.084	0.456

Values are Mean ± SD or %. hsCRP: EATV: epicardial adipose tissue volume; hsCRP: high sensitive C-reactive protein. *P1*,*P2*: P values calculated between non-CABG vs CABG and between men and women, respectively by un-paired *t* test or Fisher's exact test.

Among women, body weight, BMI, WC, and VFA were comparable between the two groups, but EATV and EATV index were higher in the CABG group. Hypertension (%), HbA1c concentration, type 2 diabetes mellitus (%), and triglyceride concentration were higher and HDL cholesterol level was lower in the CABG group. Patients with LDL cholesterol level ≥ 140 mg/dL or statin use, and hsCRP and adiponectin levels were comparable between the two groups.

### Univariate regression analysis for CABG

The univariate regression analysis to estimate the need for CABG is shown in **Table 2**. In men, CABG had significant relationships with BMI, BMI ≥ 25, VFA, EATV, and EATV index. Levels of diastolic blood pressure, plasma glucose, HbA1c, and HDL cholesterol levels, and prevalence of patients with hypertension, type 2 diabetes mellitus, LDL cholesterol level ≥140 mg/dL or statin use, and TG level ≥150 mg/dL or HDL level < 40 mg/dL were also significantly correlated to CABG. CABG was also associated with hsCRP and adiponectin levels.

**Table 2:** Univariate analysis to estimate operation of CABG.

Parameters	All (n = 172)		Men (n = 115)		Women (n = 57)	
	r	*P*	r	*P*	r	*P*
Age (years)	-0.043	0.580	-0.026	0.786	0.189	0.158
Body mass index (kg/m^2^)	0.171	0.025	0.218	0.019	-0.168	0.211
Body mass index ≥ 25 kg/m^2^ (yes/no)	0.045	0.557	0.332	0.000	-0.128	0.341
Waist circumference (cm)	0.238	0.002	0.249	0.007	0.001	0.993
Visceral fat area (cm^2^)	0.248	0.001	0.206	0.027	0.030	0.823
Subcutaneous fat area (cm^2^)	0.084	0.272	0.181	0.052	-0.062	0.646
EATV (cm^3^)	0.311	<0.0001	0.273	0.003	0.353	0.007
EATV index (cm^3^/m^2^)	0.265	0.000	0.266	0.004	0.345	0.009
Systolic blood pressure (mmHg)	0.055	0.507	0.041	0.680	0.274	0.075
Diastolic blood pressure (mmHg)	0.180	0.029	0.228	0.019	0.026	0.868
Hypertension (yes/no)	0.102	0.000	0.252	0.007	0.301	0.023
Smoking (yes/no)	0.130	0.090	0.004	0.966	0.011	0.934
Fasting plasma glucose (mg/dL)	0.176	0.032	0.276	0.005	0.133	0.377
HBA1C (%)	0.289	0.000	0.305	0.001	0.463	0.001
Type 2 diabetes mellitus (yes or no)	0.303	<0.0001	0.273	0.003	0.457	0.000
LDL cholesterol (mg/dL)	-0.092	0.247	-0.047	0.623	-0.026	0.858
LDL cholesterol ≥ 140 mg/dL or statin use (yes/no)	0.233	0.002	0.279	0.003	0.212	0.113
HDL cholesterol (mg/dL)	-0.262	0.001	-0.301	0.002	-0.378	0.006
Triglyceride (mg/dL)	0.239	0.002	0.182	0.055	0.276	0.040
Triglycerides ≥ 150 mg/dL or HDL-cholesterol < 40 mg/dL (yes/no)	0.283	0.000	0.290	0.002	0.211	0.119
hsCRP (mg/dL)	-0.091	0.235	-0.185	0.047	0.115	0.400
Adiponectin (μg/ml)	-0.232	0.002	-0.197	0.036	-0.235	0.084

EATV: epicardial adipose tissue volume; hsCRP: high sensitive C-reactive protein. r and P were calculated by univariate regression analysis to estimate operation of CABG (yes or no).

In women, CABG was not correlated with body weight, BMI, BMI ≥25, WC, and VFA, diastolic blood pressure, hsCRP levels, adiponectin levels, and prevalence of patients with LDL cholesterol level ≥140 mg/dL or statin use (%). Meanwhile, CABG was correlated with EATV, EATV index, HbA1c levels, HDL cholesterol levels, and prevalence of hypertension and type 2 diabetes mellitus.

In men, EATV index (r = 0.186, P = 0.046), as well as BMI (r = 0.337, P<0.001, data not shown) and VFA (r = 0.252, P = 0.007, data not shown), was positively correlated with Gensini score (**S1 Fig**). In women, only EATV index (r = 0.363, P = 0.006), but not BMI nor VFA, was positively correlated with Gensini score.

### Multivariate regression analysis for CABG

Next, we determined the impact of individual risk factors on CABG by multivariate regression models in a standard sequential (hierarchical) fashion (**Table 3**). In men, BMI (model 1) was a determinant of CABG after correcting for age and smoking status. Addition of hypertension, dyslipidemia (LDL cholesterol level ≥140 mg/dL or statin use; TG level ≥ 150 mg/dL or HDL cholesterol level < 40 mg/dL), and type 2 diabetes mellitus increased the corrected R^2^, which reached 0.065, 0.108 and 0.159, respectively (models 2–4). When added to the combination model using these traditional risk factors (model 1–4), VFA, the marker of visceral fat obesity, did not increase the corrected R^2^, (model 5), but addition of EATV index increased the corrected R^2^ (0.178, model 6). The addition of hsCRP and adiponectin as variables further increased the corrected R^2^ (0.256, model 7). In women, the combination model using the traditional risk factors was a significant model, but type 2 diabetes mellitus was the sole significant determinant (corrected R^2^ = 0.217, p = 0.005, model 4). Addition of VFA, EATV index, HsCRP and adiponectin as variables did not significantly improve the model (model 5–7).

**Table 3:** Multivariate analysis to estimate operation of CABG.

**Men (n = 115)**														
	Model 1		Model2		Model3		Model4		Model5		Model6		Model7	
Corrected R^2^	0.022		0.065		0.108		0.159		0.152		0.178		0.256	
*P*	0.141		0.022		0.004		0.000		0.001		0.000		0.000	
Parameters	r	*P*	r	*P*	r	*P*	r	*P*	r	*P*	r	*P*	r	*P*
Age(years)	0.016	0.864	-0.049	0.615	-0.022	0.818	-0.037	0.690	-0.037	0.687	-0.093	0.326	-0.066	0.471
Smoking status (yes or no)	-0.006	0.945	0.004	0.962	0.047	0.605	0.054	0.543	0.052	0.559	0.086	0.334	0.054	0.530
Body mass index (kg/m^2^)	0.221	0.021	0.170	0.075	0.127	0.180	0.095	0.305	0.081	0.510	0.007	0.957	-0.056	0.647
Hypertension (yes or no)			0.236	0.015	0.213	0.025	0.210	0.023	0.207	0.027	0.194	0.036	0.227	0.012
Dyslipidemia (yes or no)					0.234	0.013	0.227	0.014	0.228	0.014	0.234	0.010	0.221	0.011
Type 2 diabetes mellitus (yes or no)							0.240	0.007	0.237	0.009	0.242	0.007	0.261	0.003
Visceral fat area (cm^2^)									0.021	0.866	-0.042	0.738	-0.053	0.660
EATV index (cm^3^/m^2^)											0.225	0.036	0.265	0.011
hsCRP (mg/dL)													-0.291	0.001
Adiponectin(μg/mL)													-0.192	0.028
**Women (n = 57)**														
	Model 1		Model2		Model3		Model4		Model5		Model6		Model7	
Corrected R^2^	0.009		0.060		0.105		0.217		0.230		0.259		0.266	
*P*	0.328		0.124		0.057		0.005		0.005		0.003		0.006	
Parameters	r	*P*	r	*P*	r	*P*	r	*P*	r	*P*	r	*P*	r	*P*
Age(years)	0.180	0.185	0.119	0.381	0.081	0.544	0.095	0.446	0.120	0.341	0.067	0.594	0.072	0.589
Smoking status (yes or no)	0.063	0.646	0.074	0.579	0.018	0.892	-0.017	0.891	-0.005	0.967	-0.009	0.941	0.017	0.890
Body mass index (kg/m^2^)	-0.164	0.235	-0.148	0.272	-0.190	0.156	-0.063	0.635	-0.203	0.228	-0.200	0.226	-0.165	0.325
Hypertension (yes or no)			0.264	0.054	0.278	0.039	0.219	0.083	0.218	0.083	0.145	0.265	0.148	0.262
Dyslipidemia (yes or no)					0.253	0.064	0.170	0.191	0.163	0.207	0.169	0.182	0.118	0.380
Type 2 diabetes mellitus (yes or no)							0.373	0.006	0.356	0.008	0.341	0.010	0.370	0.011
Visceral fat area (cm^2^)									0.211	0.183	0.133	0.411	0.116	0.503
EATV index (cm^3^/m^2^)											0.230	0.094	0.186	0.217
hsCRP (mg/dL)													0.093	0.516
Adiponectin(μg/mL)													-0.163	0.208

EATV: epicardial adipose tissue volume; hsCRP: high sensitive C-reactive protein. r and P were calculated by multivariate regression analysis to estimate operation of CABG.

We further evaluated the EATV prediction models by separating the participants to groups of less visceral fat accumulation (VFA<100 cm^2^) and excess visceral fat accumulation (VFA≥100 cm^2^) (**S3 Table**). In men VFA≥100 cm^2^, EATV index, as well as hypertension, type 2 diabetes mellitus and hsCRP, was also a significant determinant for CABG. However, in men VFA<100 cm^2^, only hyperlipidemia, not EATV index, was a significant determinant. In women VFA≥100 cm^2^, there were no significant predictors, however in women VFA≥100 cm^2^, type 2 diabetes mellitus and ETV index were significant predictors.

## Discussion

The major findings of this study are: 1) in men, EATV index, in addition to traditional ASCVD risk factors including age, smoking, obesity, hypertension, type 2 diabetes mellitus, and dyslipidemia, was a significant determinant for CABG; 2) in women, meanwhile, type 2 diabetes mellitus was the sole determinant for CABG in women. EATV index as well as traditional risk factors of ASCVD were not significant determinants for CABG.

Although the prevalence of traditional ASCVD risk factors and their differential impact in women has been previously recognized [1–5], this study quantitatively determined the impact of traditional and non-traditional ASCVD risk factors in patients undergoing CABG, and discovered a large gender-specific difference.

### Gender-specific difference in traditional risk factors for CABG

The current study indicated gender-specific differences in the prevalence and quantitative impact of traditional ASCVD risk factors in patients undergoing CABG, who had severe obstructive CAD. In previous studies estimating gender-specific difference in preoperative risk factors of CABG [17–19], women were older and were diabetic, had hypercholesterolemia and hypertension, and had smaller body surface areas, as compared to men. It was also reported that women demonstrated greater 30-day mortality and late mortality than men on univariate analysis, but not on multivariate analysis after correction of preoperative risk factors [17–19]. When comparisons were made between men and women, our study also showed that women were older, had more diabetes, and had smaller body mass indices (*P*2, **Table 1**). However, when comparisons were made between CABG and non-CABG groups, the prevalence of traditional risk factors such as aging, hypertension, and dyslipidemia were significantly different in men but not in women, except for type 2 diabetes mellitus (*P*1, **Table 1**). In addition, the impact of risk factors of CABG showed gender-specific results: in men, traditional ASCVD risk factors, such as age, smoking, obesity, hypertension, diabetes mellitus, and dyslipidemia, concurrently determined the need for CABG, while in women, diabetes mellitus was the only risk factor that did.

Reportedly, the prevalence and impact of traditional risk factors for ASCVD differ between men and women [4]. CAD can be defined as vascular disease limited to the epicardial coronary arteries, and is not always the same as ischemic heart disease (IHD), which also includes ischemic disease via abnormal coronary reactivity [20] or altered microcirculation [21] in patients with non-obstructive CAD. It has been suggested that such women-specific IHD pathophysiology is associated with poor ACVSD outcomes in women [1–3].

Type 2 diabetes mellitus has long been recognized to convene a greater risk for ASCVD mortality in women than in men [22]. Juutilainen et al. reported that women with type 2 diabetes mellitus had a higher adjusted hazard ratio (HR) of fatal CAD (hazard ratio = 14.7) compared to diabetic men (hazard ratio = 3.8) [23]. A meta-analysis of 447,064 individuals confirmed that diabetic women had a 46% excess risk for fatal CAD than non-diabetic women did, compared to a smaller risk in diabetic and non-diabetic men (multiple risk-adjusted relative risks of diabetic vs non-diabetics for fatal CAD: men 1.99 vs women 3.12, p = 0.008) [24]. The Society of Thoracic Surgeons National Adult Cardiac Surgery Database (STS NCD) and the European System for Cardiac Operative Risk Evaluation (EuroSCORE) risk models independently have proposed estimation models of multiple preoperative risk factors for postoperative outcomes after cardiac surgery [25, 26]. Among the preoperative risk factors, diabetes mellitus may be more significant for women than for men [17–19]. The women-specific IHD pathophysiology such as abnormal coronary reactivity [20], altered microcirculation [21], impaired endothelium-dependent vasodilation [27], a hypercoagulable state [28], worsened atherogenic dyslipidemia [29], and vulnerability to metabolic and vascular risks [30], are proposed as possible mechanisms for poor outcomes in women with ASCVD. These mechanisms may also be associated with CABG, but evidence for the mechanisms is fairly lacking.

### Gender-specific difference in non-traditional risk factors for CABG

Among traditional ASCVD risk factors, obesity and being overweight are becoming more prevalent in the world, and significantly affect the onset of ASCVD [6]. We also employed non-traditional risk factors, including distribution in VFA [8], non-abdominal fat (EATV and EATV index) [9], and biomarkers such as CRP [15, 31] and adiponectin [15, 32],

In men, multivariate logistic regression analysis combining traditional and non-traditional risk factors showed that multiple risk factors including EATV index, hypertension, dyslipidemia, adiponectin, HsCRP, type 2 diabetes mellitus (Corrected R^2^ = 0.262, p < 0.0001) were determinants for CABG. In women, type 2 diabetes mellitus is a single strong predictor for CABG, even after addition of all traditional and non-traditional factors (Corrected R^2^ = 0.266, p = 0.005).

In 3,086 healthy participants from the Framingham Heart Study (49% women; mean age of 50 years) [7], the incidence of cardiovascular disease was associated with VFA (hazard ratio: 1.44 by a 1SD increase; p = 0.014) and non-abdominal fat (periaortic fat) (hazard ratio: 1.31; p = 0.03), but not with subcutaneous fat (hazard ratio: 0.99; p = 1.0). Since the associations were obtained even after corrections with traditional ASCVD risk factors (age, systolic blood pressure, diabetes, total cholesterol, high-density lipoprotein cholesterol, current smoking, hypertension treatment, and BMI), the abdominal and non-abdominal fat were shown to increase the prevalence and incidence of ASCVD, independent of traditional ASCVD risk factors [7, 13]. In agreement with the study, our model showed that epicardial fat volume was a significant determinant for CABG, even after correction with other traditional ASCVD risk factors.

Since it has been considered that EAT volume is highly correlated with accumulation of VAT [11], the dominant phenotype in men, we investigated parameters to estimate accumulation of EATV in men and women (**S1 and S2 Tables**). The multivariate regression analysis showed that age, VFA, CABG, and hypertension were the determinants for EATV index in men and VFA, hypertension and hsCRP were the determinants in women (**S1 Table**). Interestingly, CABG was the independent determinant for EATV index in men, but not in women. Since the biology and pathophysiological role of EAT remain unclear [9, 11, 12], we currently cannot speculate whether accumulation of EAT is a cause or a result of coronary atherosclerosis. However, our result indicated that increased EATV in men conveys an independent and additive risk when VAT mass is accounted for, suggesting that the impact of EATV is greater in men.

Our multivariate model indicated that CRP, a marker of chronic inflammation [31], and low adiponectin, a marker of adipose tissue dysfunction [8], were also independent determinants. Again, the associations of these 2 markers to CABG were observed only in men, suggesting these non-traditional ASCVD risk factors affect males dominantly. The role of chronic inflammation in the propagation of atherosclerosis and susceptibility to cardiovascular events is well established [15, 31], and hsCRP has received the most attention for its use in screening and risk classification, both in men and women [33]. We do not have any evidence to explain the gender-specific difference on the impact of hsCRP in the current study. It is known that hsCRP levels are higher among women than men and increase with age [31]. In our results, women subjects were older and had hsCRP levels than men in the CABG group, and that may affect the determinant power of hsCRP, at least partially. In the healthy lean state, adiponectin is released by EAT to decrease contractile responses to vasoconstrictive agents, thus exerting a protective anti-hypertensive function via the control of vasodilation [34]. By contrast, dependently of EATV accumulations, adiponectin was downregulated, and macrophages and proinflammatory adipocytokines were upregulated, in EAT biopsies from coronary artery disease (CAD) patients [10]. Also in EAT of heart failure (HF) patients, a regulator of adipose tissue inflammation, p53, was increased, inversely correlated with adiponectin [35]. Combined above, EAT may exert a protecting role of vascular function in healthy subjects, however, the expansion in cardiovascular diseases such as CAD and HF may lead to the development of a pro-inflammatory profile [36].

Although there have been contradictory results, a recent meta-analysis indicated a strong association between hypoadiponectinemia and cardiovascular mortality in populations with or without prior cardiovascular diseases [10][37]. In contrast, the relationship between adiponectin level and cardiovascular mortality was not observed in diabetic patients [38]. It is well-known that circulating adiponectin levels are higher in women despite their higher adiposity, which is associated in both sexes with lower adiponectin levels [39]. Even in patients after acute myocardial infarction, levels of adiponectin cannot estimate future MACE in women (best cutoff value 8.5 μg/mL, p = 0.519), while these levels can estimate MACE in men (3.8 μg/mL, p = 0.0016) [40]. Gender-specific variability in adipocyte-gene expression of adipocytokines is found for adiponectin and various other adipocyte-derived proteins, and may contribute to gender-specific adipocyte-depot distribution [41–43]. Although we previously observed that imbalance of cytokine/adipocytokine signals in EAT strongly correlated with CAD [10], this phenomenon might be limited in men.

We further evaluated gender difference in the EATV prediction models by comparing similar visceral fat distribution. In men VFA≥100 cm^2^, EATV index, hypertension, type 2 diabetes mellitus and hsCRP, were determinants for CABG. However, in men VFA<100 cm^2^, only hyperlipidemia, not EATV index, was a determinant (**S3 Table**). In women VFA≥100 cm^2^, type 2 diabetes mellitus and ETV index were predictors (**S3 Table**). These results suggest that EAT accumulation is a useful predictor for CABG in men and women, but it can differently affect coronary atherosclerosis in men and women. It might be supported by the fact that coronary atherosclerosis severity (Gensini score) was differently correlated with adiposity in men (EATV index, BMI and VFA) and women (EATV index) (**S1 Fig**).

### Study limitations

The limitations of our study arise mainly from its observational nature. Since the decision to select patients and the use of treatment or medications for ASCVD risk factors were not controlled, patient selection bias is likely present: ages were comparable between men in the non-CABG and CABG groups, but not between women. Second, our sample size is relatively small; particularly, the number of female subjects undergoing CABG was too small compared to the number of male subjects when the study population was divided into subgroups. Thus, we need to be careful when comparing men and women. In spite of these limitations, we were able to obtain similar observations with previous studies for the association of traditional ASCVD risk factors in men. Our strategy may eliminate the bias attributed to confounding factors by comparing patients of the same gender in the non-CABG and CABG groups. Some of the results of the study were not as expected, i.e. in women, the absence of association of traditional ASCVD risk factors such as age. It is ideal that more female subjects be included, to achieve adequate statistical power. Third, we selected the patients with non-coronary valvular surgery (non-CABG group) as the control, and they were not healthy population, showing increases in prevalence of hypertension and age. Shmilovich et al. determined EATV index (cm^3^ /m^2^) in healthy, low Framingham risk score people (52 ± 9 years old, 51% Male, and BMI 26.8 ± 4.9 kg/m^2^) [44]: median, range, and 25th and 75th-percentiles of EATV index were 33, 11–97, and 25 and 46 cm^3^/m^2^. EATV index of our valvular surgery controls were 53 ± 35 cm^3^/m^2^ in men and 60 ± 42 cm^3^/m^2^ in women, indicating higher values as compared to those of healthy controls [44]. Since EATV index is increased extensively with age [44, 45], we cannot simply compare theirs (mean 52 years) and ours (non-CABG/CABG 67/66 in men, 72/ 76 in women). However, since CABG groups and valvular surgery controls were eventually equalized for age and hypertension, we could minimize its confounding effects when comparing these groups.

## Conclusions

Our study revealed that multiple risk factors, including traditional risk factors and non-traditional risk factors such as epicardial fat accumulation as well as high CRP and low adiponectin levels, are determinants for CABG in men, but diabetes mellitus was the sole determinant in women. Gender disparities in ASCVD risk factors for CABG prompt us to evaluate new diagnostic and treatment strategies and to seek potential underlying mechanisms, such as epicardial fat involvement with chronic inflammation and/or hypoadiponectinemia.

## Supporting information

S1 Fig Linear correlation between epicardial adipose tissue volume (EATV) index and Gensini score in 115 men and 57 women who underwent non-coronary artery bypass graft (non-CABG: ○) or CABG (●) surgery. Linear regression analysis was made in a combined group including non-CABG and CABG subjects. R and p values are shown. In men, EATV index (r = 0.186, P = 0.046), as well as BMI (r = 0.337, P<0.001, data not shown) and VFA (r = 0.252, P = 0.007, data not shown), was positively correlated with Gensini score. In women, only EATV index (r = 0.363, P = 0.006), but not BMI nor VFA, was positively correlated with Gensini score.
(TIF)

S1 Table Univariate regression analysis between parameters. (PDF)

S2 Table Multivariate analysis to estimate EATV index. (PDF)

S3 Table Multivariate analysis to estimate operation of CABG in patients with VFA≥100 cm^2^ or with VFA<100 cm^2^. (PDF)

## Abbreviations

EATV: epicardial adipose tissue volume
CABG: coronary artery bypass grafting
ASCVD: atherosclerotic cardiovascular disease
hsCRP: high sensitive C-reactive protein
EAT: epicardial adipose tissue
CAD: coronary artery disease
VFA and SFA: visceral and subcutaneous fat area
MDCT: multi-detector computed tomography
LDL: low-density lipoprotein
TG: triglyceride
HDL: high-density lipoprotein
HbA_1c_: glycosylated hemoglobin HbA_1c_
JDS: Japan Diabetic Society
NGSP: National Glycohemoglobin Standardization Program
CT: computed tomography
BMI: body mass index
WC: waist circumference
IHD: ischemic heart disease
VAT: visceral adipose tissue
MACE: major adverse cardiac events
