# Supplementary figures and images for: Gender-linked impact of epicardial adipose tissue volume in patients who underwent coronary artery bypass graft surgery or non-coronary valve surgery
Source: PLoS One. 2017 Jun 8;12(6):e0177170. doi: 10.1371/journal.pone.0177170 (PMC5464529; doi:10.1371/journal.pone.0177170)

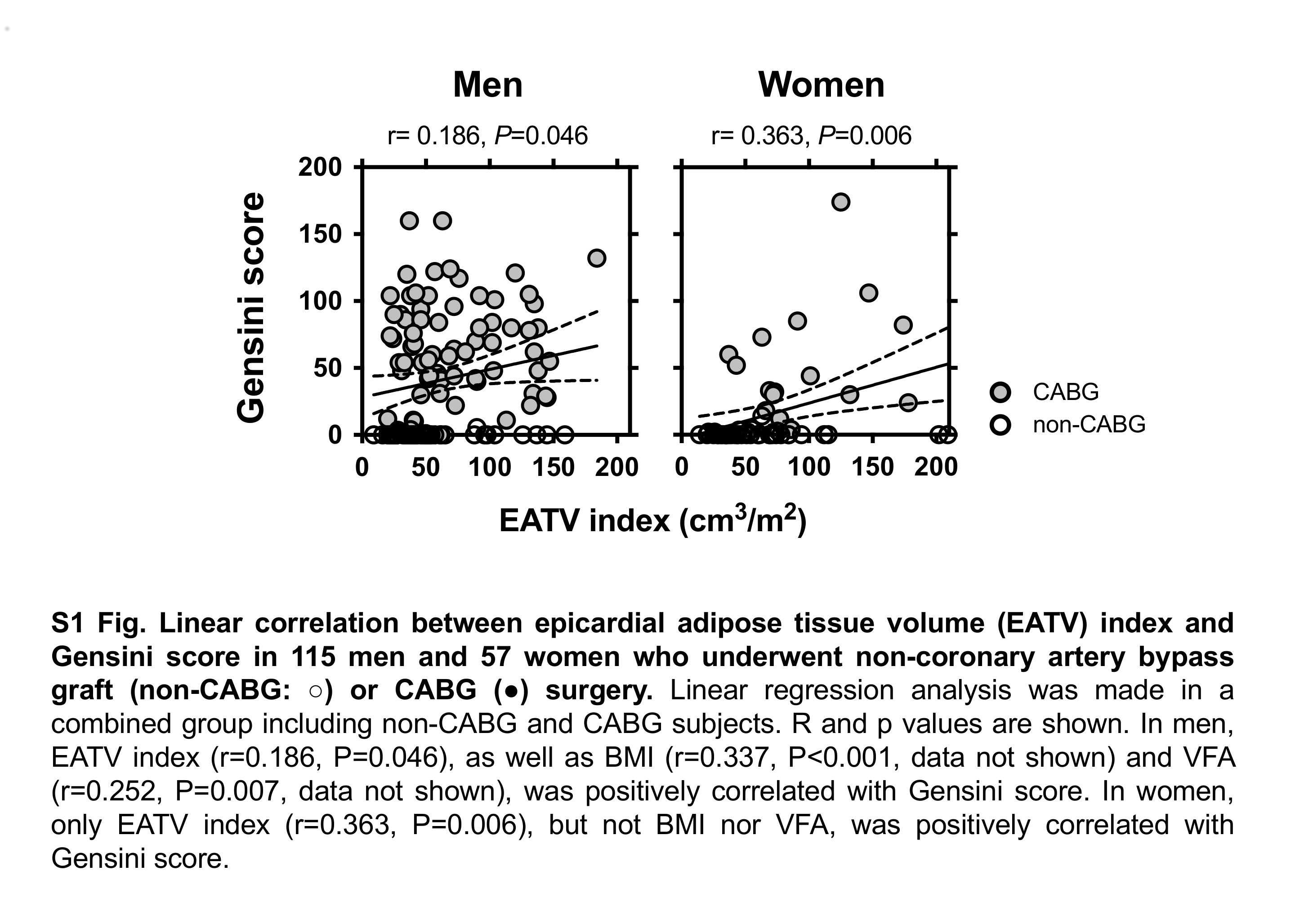

Supplement: S1 Fig — Linear regression analysis was made in a combined group including non-CABG and CABG subjects. R and p values are shown. In men, EATV index (r = 0.186, P = 0.046), as well as BMI (r = 0.337, P<0.001, data not shown) and VFA (r = 0.252, P = 0.007, data not shown), was positively correlated with Gensini score. In women, only EATV index (r = 0.363, P = 0.006), but not BMI nor VFA, was positively correlated with Gensini score. (TIF) [file pone.0177170.s001.tif]
