# Supplementary material for: Gender-linked impact of epicardial adipose tissue volume in patients who underwent coronary artery bypass graft surgery or non-coronary valve surgery
Source: PLoS One. 2017 Jun 8;12(6):e0177170. doi: 10.1371/journal.pone.0177170 (PMC5464529; doi:10.1371/journal.pone.0177170)
Supplement: S1 Table — (PDF) [file pone.0177170.s002.pdf]

S1 Table. Univariate regression analysis between parameters

Men (n=115〕

Parameters	CABG	Age	Visceral fat area	EATV index	Hypertension	Dyslipidemia	Diabetes Mellitus	C Reactive protein	Adiponectine	Smoking status
CABG (yes or no)	1									
Age (years)	-0.026	1								
Visceral fat area (cm^2)	0.206*	-0.062	1							
EATV index (cm^3/m^2)	0.266**	0.196*	0.477***	1						
Hypertension (yes or no)	0.252**	0.236*	0.224*	0.236*	1					
Dyslipidemia (yes or no)	0.279**	-0.112	0.090	0.069	0.112	1				
Diabetes Mellitus(yes or no)	0.273**	0.039	0.234*	0.100	0.054	0.057	1			
C Reactive protein (mg/dL)	-0.185*	0.178	-0.009	0.134	0.127	-0.067	0.173	1		
Adiponectine(μg/mL)	-0.197*	0.075	-0.128	-0.037	0.027	0.049	-0.152	-0.089	1	
Smoking status (yes or no)	0.004	-0.076	0.106	-0.121	-0.055	-0.169	-0.031	-0.042	-0.196*	1

Women (n=57)

Parameters	CABG	Age	VFA	EATV Index	Hypertension	Hyperlipidemia	T2DM	CRP	Adiponectin	smoking
CABG (yes or no)	1									
Age (years)	0.189	1								
Visceral fat area (cm^2)	0.030	-0.164	1							
EATV index (cm^3/m^2)	0.345**	0.248*	0.265*	1						
Hypertension (yes or no)	0.301*	0.243	-0.068	0.364**	1					
Dyslipidemia (yes or no)	0.212	0.097	0.157	0.044	-0.052	1				
Diabetes Mellitus(yes or no)	0.458***	0.045	-0.114	0.088	0.160	0.163	1			
C Reactive protein (mg/dL)	0.115	0.311	-0.177	0.295	0.126	0.077	0.222	1		
Adiponectine(μg/mL)	-0.235	0.118	-0.249	-0.055	-0.005	-0.222	-0.057	-0.072	1	
Smoking status (yes or no)	0.011	-0.100	0.104		-0.079	0.246	0.071	-0.087	0.071	1

EATV: epicardial adipose tissue volume; CABG: coronary artery bypass graft. r and P were calculated by univariate regression analysis.
