# Supplementary material for: Gender-linked impact of epicardial adipose tissue volume in patients who underwent coronary artery bypass graft surgery or non-coronary valve surgery
Source: PLoS One. 2017 Jun 8;12(6):e0177170. doi: 10.1371/journal.pone.0177170 (PMC5464529; doi:10.1371/journal.pone.0177170)
Supplement: S2 Table — (PDF) [file pone.0177170.s003.pdf]

S2 Table. Multivariate analysis to estimate EATV Index

Men (n=115)

	Model 1		Model 2		Model 3		Model 4		Model 5		Model 6		Model 7		Model 8		Model 9	
Corrected R^2	0.265		0.290		0.285		0.278		0.276		0.293		0.264		0.296		0.300	
P	<0.0001		<0.0001		<0.0001		<0.0001		<0.0001		<0.0001		<0.0001		<0.0001		<0.0001	
Parameters	**r**	**P**	**r**	**P**	**r**	**P**	**r**	**p**	**r**	**p**	**r**	**p**	**r**	**p**	**r**	**p**	**r**	**p**
Age(years)	0.226	0.006	0.228	0.005	0.218	0.009	0.218	0.010	0.223	0.009	0.204	0.016	0.217	0.012	0.211	0.012	0.188	0.028
Visceral fat area (cm^2)	0.491	<0.0001	0.454	<0.0001	0.446	<0.0001	0.446	<0.0001	0.460	<0.0001	0.466	<0.0001	0.467	<0.0001	0.482	<0.0001	0.493	<0.0001
CABG (yes or no)	-	-	0.178	0.029	0.169	0.044	0.169	0.053	0.186	0.039	0.232	0.012	0.184	0.046	0.196	0.028	0.240	0.011
Hypertension (yes or no)	-	-	-	-	0.042	0.627	0.042	0.630	0.037	0.672	0.010	0.906	0.033	0.713	0.028	0.749	-0.002	0.981
Dyslipidemia (yes or no)	-	-	-	-	-	-	0.002	0.984	0.001	0.994	0.001	0.988	-0.010	0.911	-0.031	0.710	-0.037	0.660
Diabetes Mellitus(yes or no)	-	-	-	-	-	-	-	-	-0.069	0.415	-0.109	0.209	-0.072	0.408	-0.079	0.345	-0.120	0.168
C Reactive protein (mg/dL)	-	-	-	-	-	-	-	-	-	-	0.162	0.059	-	-	-	-	0.163	0.061
Adiponectine(μg/mL)	-	-	-	-	-	-	-	-	-	-	-	-	0.031	0.712	-	-	0.027	0.749
Smoking status (yes or no)	-	-	-	-	-	-	-	-	-	-	-	-	-	-	-0.163	0.047	-0.154	0.066

Women (n=57)

	Model 1		Model 2		Model 3		Model 4		Model 5		Model 6		Model 7		Model 8		Model 9	
Corrected R^2	0.126		0.195		0.250		0.239		0.224		0.293		0.218		0.209		0.272	
P	0.0098		0.0023		0.0007		0.0018		0.0041		0.001		0.0083		0.0085		0.0041	
Parameters	**r**	**p**	**r**	**p**	**r**	**p**	**r**	**p**	**r**	**p**	**r**	**p**	**r**	**p**	**r**	**p**	**r**	**p**
Age(years)	0.300	0.022	0.242	0.057	0.191	0.123	0.198	0.115	0.196	0.125	0.133	0.292	0.199	0.132	0.198	0.126	0.124	0.346
Visceral fat area (cm^2)	0.314	0.016	0.295	0.019	0.308	0.012	0.318	0.011	0.313	0.014	0.386	0.003	0.330	0.013	0.312	0.016	0.400	0.003
CABG (yes or no)	-	-	0.290	0.022	0.217	0.083	0.230	0.075	0.243	0.091	0.153	0.280	0.245	0.106	0.244	0.093	0.183	0.217
Hypertension (yes or no)	-	-	-	-	0.274	0.032	0.265	0.040	0.267	0.041	0.258	0.041	0.271	0.043	0.267	0.043	0.254	0.049
Dyslipidemia (yes or no)	-	-	-	-	-	-	-0.060	0.624	-0.057	0.647	-0.110	0.369	-0.082	0.531	-0.063	0.628	-0.115	0.382
Diabetes Mellitus(yes or no)	-	-	-	-	-	-	-	-	-0.029	0.830	0.118	0.408	0.002	0.990	-0.031	0.823	0.119	0.411
C Reactive protein (mg/dL)	-	-	-	-	-	-	-	-	-	-	0.307	0.022	-	-	-	-	0.314	0.023
Adiponectine(μg/mL)	-	-	-	-	-	-	-	-	-	-	-	-	0.045	0.733	-	-	0.077	0.551
Smoking status (yes or no)	-	-	-	-	-	-	-	-	-	-	-	-	-	-	0.023	0.852	0.016	0.895

EATV: epicardial adipose tissue volume; CABG: coronary artery bypass graft. r and P were calculated by multivariate regression analysis to estimate EATV index.
