# Supplementary material for: Gender-linked impact of epicardial adipose tissue volume in patients who underwent coronary artery bypass graft surgery or non-coronary valve surgery
Source: PLoS One. 2017 Jun 8;12(6):e0177170. doi: 10.1371/journal.pone.0177170 (PMC5464529; doi:10.1371/journal.pone.0177170)
Supplement: S3 Table — (PDF) [file pone.0177170.s004.pdf]

S3 Table. Multivariate analysis to estimate operation of CABG in patients with VFA≥100 cm^2 or with VFA<100 cm^2

Men (n= 82; VFA≥100 cm^2〕

	Model 1		Model 2		Model 3		Model 4		Model 5		Model 6		Model 7	
Adjusted R^2	0.004		0.087		0.087		0.161		0.149		0.167		0.239	
P	0.349		0.026		0.035		0.004		0.007		0.005		0.001	
Parameters	r	*P*	r	*P*	r	*P*	r	*P*	r	*P*	r	*P*	r	*P*
Age (years)	0.140	0.231	0.074	0.513	0.085	0.458	0.036	0.745	0.036	0.748	-0.036	0.766	-0.035	0.764
Smoking status (yes or no)	-0.079	0.480	-0.058	0.587	-0.031	0.776	0.001	0.994	0.001	0.993	0.052	0.642	0.082	0.456
Body mass index (kg/m^2)	0.169	0.148	0.127	0.257	0.111	0.328	0.079	0.471	0.079	0.563	0.029	0.834	0.055	0.689
Hypertension (yes or no)			0.310	0.006	0.302	0.007	0.299	0.006	0.299	0.007	0.296	0.007	0.366	0.001
Dyslipidemia (yes or no)					0.112	0.319	0.113	0.296	0.113	0.299	0.124	0.252	0.046	0.672
Diabetes Mellitus(yes or no)							0.288	0.007	0.288	0.009	0.303	0.006	0.353	0.001
Visceral fat area (cm^2)									-0.001	0.996	-0.097	0.522	-0.224	0.156
EATV index (cm^3/m^2)											0.214	0.112	0.335	0.019
C Reactive protein (mg/dL)													-0.286	0.008
Adiponectine(μg/mL)													-0.169	0.147

Men (n= 33; VFA<100〕

	Model 1		Model 2		Model 3		Model 4		Model 5		Model 6		Model 7	
Corrected R^2	0.083		0.053		0.294		0.267		0.239		0.236		0.294	
P	0.141		0.246		0.012		0.025		0.047		0.061		0.047	
Parameters	r	*P*	r	*P*	r	*P*	r	*P*	r	*P*	r	*P*	r	*P*
Age (years)	-0.166	0.350	-0.185	0.341	-0.124	0.461	-0.127	0.463	-0.129	0.464	-0.165	0.364	-0.063	0.745
Smoking status (yes or no)	0.064	0.720	0.066	0.717	0.106	0.501	0.109	0.502	0.106	0.525	0.120	0.474	0.127	0.432
Body mass index (kg/m^2)	0.324	0.074	0.309	0.109	0.167	0.325	0.166	0.339	0.189	0.356	0.118	0.587	0.126	0.554
Hypertension (yes or no)			0.051	0.790	-0.025	0.882	-0.024	0.888	-0.019	0.914	-0.036	0.839	-0.035	0.837
Dyslipidemia (yes or no)					0.516	0.003	0.518	0.004	0.514	0.004	0.518	0.005	0.513	0.005
Diabetes Mellitus(yes or no)							-0.021	0.892	-0.023	0.884	-0.015	0.925	0.044	0.786
Visceral fat area (cm^2)									-0.043	0.820	-0.040	0.834	-0.058	0.774
EATV index (cm^3/m^2)											0.167	0.353	0.282	0.133
C Reactive protein (mg/dL)													-0.346	0.067
Adiponectine(μg/mL)													0.020	0.929

Women (n= 23; VFA≥100〕

	Model 1		Model 2		Model 3		Model 4		Model 5		Model 6		Model 7	
Corrected R^2	-0.140		-0.036		-0.013		0.085		0.111		0.056		-0.148	
P	0.960		0.534		0.477		0.296		0.279		0.385		0.686	
Parameters	r	*P*	r	*P*	r	*P*	r	*P*	r	*P*	r	*P*	r	*P*
Age (years)	0.003	0.992	0.021	0.936	-0.019	0.942	0.006	0.981	0.047	0.854	0.070	0.796	0.008	0.983
Smoking status (yes or no)	-0.045	0.874	0.048	0.862	-0.085	0.773	0.047	0.872	0.070	0.809	0.084	0.781	0.081	0.842
Body mass index (kg/m^2)	-0.103	0.667	-0.174	0.457	-0.171	0.461	0.072	0.785	-0.154	0.633	-0.167	0.620	-0.148	0.698
Hypertension (yes or no)			0.383	0.105	0.367	0.116	0.296	0.189	0.230	0.309	0.269	0.300	0.284	0.388
Dyslipidemia (yes or no)					0.279	0.252	0.080	0.756	0.094	0.713	0.054	0.848	0.107	0.780
Diabetes Mellitus(yes or no)							0.468	0.113	0.453	0.121	0.473	0.124	0.467	0.234
Visceral fat area (cm^2)									0.343	0.246	0.362	0.243	0.336	0.349
EATV index (cm^3/m^2)											-0.097	0.726	-0.019	0.960
C Reactive protein (mg/dL)													0.070	0.854
Adiponectine(μg/mL)													0.111	0.768

Women (n= 34; VFA<100〕

	Model 1		Model 2		Model 3		Model 4		Model 5		Model 6		Model 7	
Corrected R^2	0.026		0.022		0.040		0.162		0.159		0.399		0.343	
P	0.295		0.338		0.303		0.091		0.112		0.005		0.030	
Parameters	r	*P*	r	*P*	r	*P*	r	*P*	r	*P*	r	*P*	r	*P*
Age (years)	0.253	0.155	0.185	0.333	0.127	0.516	0.207	0.269	0.232	0.223	0.167	0.300	0.121	0.514
Smoking status (yes or no)	0.086	0.635	0.096	0.600	0.070	0.698	-0.073	0.687	-0.118	0.531	-0.191	0.240	-0.110	0.547
Body mass index (kg/m^2)	-0.231	0.205	-0.223	0.222	-0.241	0.185	-0.130	0.459	-0.142	0.420	-0.150	0.316	-0.185	0.298
Hypertension (yes or no)			0.175	0.357	0.201	0.290	0.098	0.591	0.099	0.588	-0.097	0.555	-0.075	0.680
Dyslipidemia (yes or no)					0.223	0.225	0.164	0.343	0.161	0.352	0.031	0.835	0.045	0.802
Diabetes Mellitus(yes or no)							0.406	0.033	0.407	0.033	0.452	0.007	0.391	0.033
Visceral fat area (cm^2)									0.161	0.348	0.034	0.820	0.003	0.984
EATV index (cm^3/m^2)											0.549	0.002	0.524	0.014
C Reactive protein (mg/dL)													0.082	0.643
Adiponectine(μg/mL)													-0.130	0.474

EATV: epicardial adipose tissue volume; CABG: coronary artery bypass graft; VFA: visceral fat area. r and P were calculated by multivariate regression analysis to estimate CABG.
